# Pediatric Dilated Cardiomyopathy With Suspected Hereditary Protein C/S and Antithrombin III Deficiency Presenting as Ischemic Stroke—A Rare Case Report

**DOI:** 10.1155/crpe/3631063

**Published:** 2026-10-07

**Authors:** Muhammad Waleed Imran, Muhammad Moaz, Sanwal Sardar Nawaz, Amna Ilyas, Sabeen Abid

**Affiliations:** ^1^ Shifa College of Medicine Islamabad Pakistan; ^2^ Department of Pediatrics Shifa International Hospital Islamabad Pakistan shifa.com.pk

## Abstract

**Objective:**

To report a rare case of pediatric dilated cardiomyopathy (DCM) with suspected hereditary protein C, protein S, and antithrombin III deficiency presenting as ischemic stroke.

**Background:**

Children with DCM are at increased risk of thromboembolism. Inherited thrombophilias are an uncommon but important cause of pediatric stroke and may coexist with cardiac disease.

**Case Presentation:**

An 8‐year‐old female presented with heart failure and was diagnosed with severe DCM (LVEF ∼24%). Despite medical therapy and antiplatelet prophylaxis, she developed acute right‐sided hemiparesis. MRI confirmed a left hemispheric ischemic stroke. Thrombophilia workup revealed reduced protein C (41.5%), protein S (50.7%), and antithrombin III (65.1%) levels, suggestive of a suspected inherited thrombophilic disorder, while PT/aPTT/INR remained normal. She was managed conservatively with antiplatelets and rehabilitation, showing partial neurological recovery. Follow‐up demonstrated improved cardiac function (LVEF ∼50%).

**Conclusion:**

The coexistence of DCM and suspected inherited thrombophilia can predispose to ischemic stroke in children. Normal routine coagulation tests do not exclude thrombophilia; targeted screening should be considered in high‐risk cases.

## 1. Introduction

Dilated cardiomyopathy (DCM) is a heart muscle disease that includes left ventricular or biventricular dilatation and systolic dysfunction with no coronary artery disease significant enough to explain the dysfunction. DCM is relatively uncommon in children, with reported prevalence estimates ranging from 1/2500 to 1/250 [1]. Progressive systolic dysfunction may result in severe heart failure and an increased risk of adverse cardiac outcomes [2]. Multiple genetic variants have been associated with familial cardiomyopathy, with several recognized patterns of inheritance reported in affected families [3]. Severe ventricular dysfunction is associated with poorer outcomes in children with DCM [4]. Reported survival rates range from 63% to 90% at 1 year and from 20% to 80% at 5 years [5].

The Global Burden of Disease (GBD) 2019 report indicates that between 1990 and 2019, both the annual incidence of stroke and the number of stroke‐related deaths rose markedly [6]. Genetic abnormalities of the coagulation system are now being increasingly recognized as one of the important causes of stroke in children. Protein C (PC) is a vitamin K‐dependent serine protease that, when activated, inhibits thrombin production by inactivating activated factors V and VIII. Protein S (PS) acts as a cofactor, enhancing PC’s function. PC and PS are natural anticoagulants that regulate coagulation, and deficiencies in either protein are associated with an increased risk of thrombosis [7].

## 2. Case Presentation

A 5‐year‐old female was first evaluated for recurrent breath‐holding spells and failure to thrive. Developmental history was normal, and there was no history of significant infections or toxin exposure. Family history was significant for a brother diagnosed with DCM and a history of maternal pregnancy loss. Physical examination at that time revealed pallor but no overt cyanosis or clubbing. Blood tests showed mild microcytic anemia, consistent with iron deficiency (Table 1). Echocardiography revealed a structurally normal heart except for trivial to mild mitral regurgitation; ventricular size and function were essentially normal at that time. She was diagnosed with nutritional anemia and treated with iron supplementation.

**TABLE 1:** Serial complete blood count parameters over time.

Parameter	10–09‐2022	15–05‐2024	14–12‐2024	17–05‐2025	Reference range
WBC (/μL)	5260	4660	4850	6730	5000–13,000
RBC (× 10^6^/μL)	5.26	4.66	4.85	4.94	4.50–5.50
Hemoglobin (g/dL)	10.4	11.2	11.2	11.1	13.5–16.0
Hematocrit (%)	35.6	34.4	35.7	34.7	35.0–50.0
MCV (fL)	67.7	73.8	73.7	70.2	77.0–95.0
MCH (pg)	19.8	24.0	23.0	22.5	25.0–33.0
MCHC (g/dL)	29.2	32.3	31.0	32.0	31.0–37.0
Platelet count (/μL)	286,000	168,000	175,000	184,000	170,000–450,000
Neutrophils (%)	43	39	34	49	40–63
Lymphocytes (%)	38	49	48	35	20–48
Monocytes (%)	11	9	17	9	2–8
Eosinophils (%)	0	2	1	7	1–6
Basophils (%)	0	0	0	0	0–1

The patient was lost to follow‐up for 3 years. At age 8 years, she re‐presented with progressive dyspnea on exertion and swelling of the legs. On examination she appeared cachectic, with tachycardia (heart rate ∼110/min), and 2+ pitting edema in the lower extremities. Blood pressure and respiratory rate were normal. A repeat echocardiogram now showed a markedly dilated left ventricle with severely reduced systolic function (left ventricular ejection fraction ∼24%). Mild mitral and tricuspid regurgitation was present, with no intracardiac thrombi visualized. No vegetation was seen on any valve. These findings confirmed DCM.

She was admitted to the pediatric cardiology service. Medical therapy for heart failure was initiated, including intravenous diuretics followed by oral furosemide and spironolactone for volume management, as well as an ACE inhibitor (enalapril) for afterload reduction. Due to concern for thromboembolic risk in severe DCM, low‐dose aspirin (5 mg/kg/day) was started for antiplatelet prophylaxis. Hemoglobin at this admission was 9.8 g/dL (microcytic); renal and liver function tests were normal. There was no evidence of acute infection; blood cultures drawn at this time remained negative. The combination of heart failure and anemia was managed supportively.

Two months after initiating heart failure therapy (still at 8 years of age), the patient awoke with new right‐sided weakness. There was no preceding trauma or acute illness. The neurologic exam showed right hemiparesis (strength ∼ grade 4/5), increased tone on the right, and a right Babinski sign; cranial nerves were intact. An emergent brain MRI revealed an infarction in the left cerebral hemisphere consistent with acute ischemic stroke (presumed left middle cerebral artery distribution). Magnetic resonance angiography did not demonstrate large vessel occlusion or vasculitis.

An evaluation for stroke etiology was undertaken. A coagulation/thrombophilia panel was sent. Notably, PC activity was 41.5% (reference 75%–120%), and PS activity was 50.7% (63%–116%). The antithrombin III level was 65.1% (80%–120%) (Table 2). These results were confirmed on repeat testing 1 month later after recovery from the acute thrombotic event. No PC or PS mutation analysis was available due to cost constraints; however, the persistently low functional levels in multiple tests raised suspicion for the presumed hereditary deficiency, although it could not be confirmed. Screening for other causes (lipid profile, homocysteine, and sickle cell test) was unremarkable; the activated PC resistance assay was carried out to screen for Factor V Leiden mutation which also came out to be in the normal range of 1.03 (0.86–1.10) making it highly unlikely. Acquired causes of anticoagulant reduction, such as severe infection or hepatic dysfunction, were considered but deemed unlikely given the negative blood cultures and normal liver function tests; however, they could not be completely excluded. Serial PT, aPTT, and INR measurements at admission, at stroke, and at later follow‐ups all remained within normal limits (Table 3).

**TABLE 2:** Thrombophilia profile showing reduced protein C, protein S, and antithrombin III levels.

Test	Patient value (%)	Normal range (%)
Protein C	41.50	75–120
Protein S	50.70	63–116
Antithrombin III	65.10	80–120

**TABLE 3:** Coagulation profile at multiple time points.

Test	17–05‐2025	14–12‐2024	28–05‐2024	Normal range
PT (sec)	10.6	11.5	10.9	10.1–12.0
aPTT (sec)	30.80	30.40	28.30	26–36
INR	0.98	1.06	—	0.9–1.2

*Note:* Prothrombin time (PT), activated partial thromboplastin time (aPTT), and international normalized ratio (INR) remained within reference ranges, supporting the presence of abnormalities in natural anticoagulant proteins despite normal routine coagulation parameters.

She was managed conservatively for stroke with continued antiplatelet therapy. Physical and occupational therapy were initiated, with gradual improvement in right‐sided strength (at discharge, arm and leg power ∼4/5). An infantile (Osler) lesion on a toe raised concern for endocarditis, but blood cultures and echocardiograms did not support this, and thus infective endocarditis was not definitively diagnosed.

At her most recent follow‐up (at age 9 in 2025), the patient’s neurologic deficits had partially improved (mild residual right hemiparesis). Repeat echocardiography showed marked improvement in ventricular function, with LVEF ∼50%. Heart failure medications were continued. She remains on low‐dose aspirin. Therapeutic anticoagulation (e.g., warfarin) was withheld because financial limitations and concerns regarding poor follow‐up made regular INR monitoring impractical, resulting in an unacceptably high risk of bleeding. Her growth has improved modestly with nutritional support. Detailed genetic testing (for cardiomyopathy genes or PROC/PROS genes) was not performed due to financial limitations.

## 3. Discussion

Pediatric DCM, suspected hereditary coagulopathy, and subsequent ischemic stroke are key characteristics of this case. The patient’s family history of DCM in a sibling and maternal pregnancy loss raises suspicion for a familial cause. In the pediatric population, the most prevalent causes include idiopathic or familial forms. Infective endocarditis was considered because of the toe lesion; however, serial echocardiography showed no vegetations, and blood cultures remained negative, with no other evidence supporting infective endocarditis. Iron deficiency in addition to beta thalassemia trait was thought to be the cause of microcytosis and anemia; thalassemia, however, is not associated with cardiomyopathy. The presence of DCM in the patient’s brother suggests a potential familial or genetic origin for the cardiomyopathy. Nevertheless, because specific genetic screening and comprehensive family segregation analyses were not conducted, a definitive genetic cause and inheritance pattern remain unconfirmed.

The occurrence of stroke in this patient requires thorough evaluation. As reported in the GBD 2019 study, there was a significant increase in yearly stroke cases and associated mortality from 1990 to 2019 [6]. It is still considered a rare occurrence, especially in pediatric population, which results in a delay in identifying it [8]. The causes are distinct from the adult population, with predominant factors including congenital or acquired heart diseases, aortic, or cervical arteriopathy, Moyamoya disease, small vessel arteriopathy, severe thrombophilia, proinflammatory conditions, genetic vasculopathy, or infectious disorders [9]. A rare cause of arterial thrombosis and ischemic stroke is PC deficiency. PC is a vitamin K–dependent serine protease. Activated PC prevents thrombin generation by deactivating activated Factors V and VIII. This process is facilitated by PS, which acts as a cofactor for PC. PC and PS are vitamin K‐dependent natural anticoagulants that regulate coagulation, and deficiencies in either protein are associated with an increased risk of thrombosis [7]. It is noteworthy that routine coagulation parameters (PT, aPTT, and INR) were within normal limits, a finding consistent with derangements in natural anticoagulants. This highlights that normal baseline coagulation studies do not exclude thrombophilia, and specific assays are required for diagnosis. In several case reports, patients with PC or PS deficiency have had normal routine coagulation studies (PT/aPTT) [10]. Our patient suffered a left hemispheric ischemic stroke, presenting with right‐sided neurological deficits. Radiological imaging confirmed the ischemic stroke though its exact etiology could not be established. The patient’s severe DCM and sustained abnormalities in PC, PS, and antithrombin III may have increased their risk of thrombosis; however, no intracardiac thrombus or other definite embolic source was identified. The diagnosis was established using functional tests. A definitive conclusion may have been obtained through genetic testing of the Protein C gene (PROC) and Protein S1 gene (PROS1); however, this was not possible.

A study described that even in the presence of other cardiac lesions; PC deficiency may be associated with intracardiac thrombi, with four out of 11 cases reporting DCM as the predisposing condition for clot formation [11]. Another study highlights the significance of evaluating hemostatic disorders in the background of DCM [12]. In retrospect, starting prophylactic anticoagulation might have been possible if thrombophilia screening had been done during the first admission. Even though routine testing is debated because this combination is so rare, our case shows the value of keeping it in mind when pediatric DCM is diagnosed, especially if there are unexplained neurological symptoms.

The management of this patient also has key takeaways. The initiation of diuretics and ace inhibitors for heart failure resulted in significant improvement in LV function which is a positive sign as it indicates nonischemic DCM that might be reversible. Given the patient’s severe DCM, ischemic stroke, and persistently reduced PC, PS, and antithrombin III profile, therapeutic anticoagulation was considered. However, antiplatelet therapy was continued due to the absence of a documented intracardiac thrombus and the uncertain mechanism of the stroke. Additionally, financial limitations and concerns regarding reliable follow‐up made the routine INR monitoring required for warfarin impractical, which would have resulted in an unacceptably high risk of bleeding.

Reports in the literature suggest that PC deficiency may act as a precipitating factor for thrombosis in the presence of DCM [12]. While these case reports support a potential association between natural anticoagulant deficiencies and thrombotic events, a definitive causal relationship in our specific patient cannot be firmly established. Our case shows a combination of pediatric DCM and a suspected inherited PC/PS deficiency in a child that subsequently developed ischemic stroke, although a causal relationship could not be established. While each condition is rare, their overlap should make clinicians think more broadly about the diagnosis. It also underlines the value of thrombophilia screening when a child with DCM develops neurological symptoms that are not otherwise explained. In children, stroke is often missed or diagnosed late, so earlier recognition could make a real difference by allowing earlier assessment of thrombotic risk and individualized antithrombotic management.

## Funding

No funding was received for this manuscript.

## Consent

Written informed consent was obtained for the publication of this case report and any accompanying clinical and laboratory details. A copy of the written consent is available for review upon request.

## Conflicts of Interest

The authors declare no conflicts of interest.

## Data Availability

The data that support the findings of this study are available from the corresponding author upon reasonable request.
